# A novel method for quantifying axon degeneration

**DOI:** 10.1371/journal.pone.0199570

**Published:** 2018-07-18

**Authors:** Aaron D. Johnstone, Robin M. Hallett, Andrés de Léon, Bruno Carturan, Julien Gibon, Philip A. Barker

**Affiliations:** 1 Department of Neurology and Neurosurgery, Montreal Neurological Institute, McGill University, Montreal, Quebec, Canada; 2 Department of Biology, University of British Columbia Okanagan, Kelowna, British Columbia, Canada; 3 The Hospital for Sick Children, University of Toronto, Toronto, Ontario, Canada; University of Edinburgh, UNITED KINGDOM

## Abstract

Axons normally degenerate during development of the mammalian nervous system, but dysregulation of the same genetically-encoded destructive cellular machinery can destroy crucial structures during adult neurodegenerative diseases. Nerve growth factor (NGF) withdrawal from dorsal root ganglia (DRG) axons is a well-established *in vitro* experimental model for biochemical and cell biological studies of developmental degeneration. Definitive methods for measuring axon degeneration have been lacking and here we report a novel method of axon degeneration quantification from bulk cultures of DRG that enables objective and automated measurement of axonal density over the entire field of radial axon outgrowth from the ganglion. As proof of principal, this new method, written as an R script called Axoquant 2.0, was used to examine the role of extracellular Ca^2+^ in the execution of cytoskeletal disassembly during degeneration of NGF-deprived DRG axons. This method can be easily applied to examine degenerative or neuroprotective effects of gene manipulations and pharmacological interventions.

## Introduction

Neurodegeneration occurs normally during embryonic development to establish and refine the maturing nervous system. Importantly, molecular components of the same destructive signaling pathways also appear to underlie neurodegenerative diseases such as Alzheimer’s, Parkinson’s and ALS when they are aberrantly reactivated in adulthood due to interactions between genetic and environmental factors [1–4]. The emerging overlap between developmental and pathological mechanisms of neurodegeneration suggests that understanding the sequence of molecular events comprising physiological neurite degeneration and cell death programs during development will reveal therapeutic opportunities that interrupt disease.

Developmental neurodegeneration has been modeled *in vitro* by nerve growth factor (NGF) withdrawal from dorsal root ganglia (DRG) [5,6]. *In vivo*, axons of sensory neurons of DRG connect peripheral tissues with the spinal cord to establish connectivity with the central nervous system. Initially, an excess of DRG neurons are generated and the mature nervous system pattern is established by those neurons that arrive at their correct targets and receive adequate trophic support, while those that fail to target undergo cell death [1,7–10]. A majority of DRG axons respond to trophic support from target-derived nerve growth factor (NGF). NGF binds to its high-affinity surface receptor TrkA on axons to activate its cytoplasmic tyrosine kinase domain, initiating a pro-survival signaling cascade that depends on retrograde trafficking of signaling endosomes [1,3,11]. It was long assumed that the degeneration of those axons that fail to reach their targets was due simply to a deficiency in survival signaling initiated by NGF, but it is now clear that degeneration is an active, regulated process, and is reversible at several stages [12].

*In vitro*, explanted DRG ganglia extend axons onto the culture substrate in the presence of NGF and these can be induced to degenerate by NGF withdrawal and/or by application of a function-blocking antibody against NGF [5,6]. Although the neurotrophic support of DRG neurons (and their degeneration and death following trophic support withdrawal) was discovered by Nobel laureate Rita Levi-Montalcini in 1951, key aspects of the signaling process underlying degeneration remain obscure and mechanistically incomplete [6,13,14]. Elucidating the molecular pathways underpinning neurodegeneration in the normal physiological context of developmental degeneration will identify new lines of inquiry for understanding how to slow progression of adult neurodegenerative diseases.

Effective use of the DRG & NGF withdrawal system to dissect degenerative signaling *in vitro* relies on objective quantification of axon degeneration. The challenge is heightened by variability in axon length, density and morphology that open the possibility of sampling biases that mask or amplify bona fide biological effects. To address this, we have developed the first automated method for quantifying neurodegeneration from micrographs of whole DRG explants. Unlike currently existing strategies for quantifying degeneration, Axoquant 2.0 reports the degree of axon degeneration over the entire radial growth field from soma to growth cone. It avoids variability introduced by random sampling from within axon fields and circumvents the need to generate dissociated neuron cultures or any manual, subjective quantification based on qualitative criteria that are time consuming and vulnerable to bias. Axoquant 2.0 is written in R, a programming language familiar to computational biologists, bioinformaticians and statisticians, but easily accessible to the first-time user when deployed with the open-ware graphical user interface, R-Studio (http://www.rstudio.com). Additionally, our method can be applied to other models of neurodegeneration that utilize DRG cultures such oxygen/glucose deprivation, oxidative stress or chemotherapeutic-induced neurodegeneration.

As proof-of-principle, we show that Axoquant 2.0 reveals striking preservation of the tubulin cytoskeleton in DRG axons by Ca^2+^ chelator EGTA, providing previously unreported direct evidence of a role for Ca^2+^ in developmental neurodegeneration.

## Material and methods

### Preparation, culture and immunocytochemical staining of DRG explants

DRG explant culture, fixation and immunostaining was performed on CD1 embryos (Charles River Laboratories) at 13.5 days post-fertilization as previously described [15] except that here DRG explants were grown on 6-well plastic plates (Greiner bio-one). All experimental procedures were approved by the Montreal Neurological Institute Animal Care Committee and University of British Columbia animal care committees and were in compliance with Canadian Council on Animal Care guidelines.

### Ca^2+^ chelation by EGTA

DRG explants were seeded on 6-well plastic cell culture plates (Greiner bio-one) in media containing 12.5 ng/ml NGF (Alomone). After 60h of growth in NGF, cultures were either maintained in NGF or were deprived of NGF and exposed to anti-NGF antibody (2.8 ug/ml) in the presence of EGTA 6 mM (Alfa Aesar) for the 24 hour duration of NGF withdrawal.

### Imaging and image pre-processing

DRG cultures fixed in 4% paraformaldehyde in PBS and immunostained with mouse anti-β-III tubulin (Millipore; 1:10 000) primary antibody and anti-mouse secondary conjugated to Alexa Fluor 488 (ThermoFisher; 1:5 000) were imaged at 5x magnification using a Zeiss Axioscope2 inverted epifluorescence microscope with an automated, motorized stage. Images were stitched automatically with Zen 2 software from Zeiss to produce a master image of all explants on the entire 6-well plate. From this master image, quarter-DRG fields were cropped using NIH ImageJ (FIJI build) to create an image set for quantification.

### Quantification of axon area with Axoquant 2.0 in R Studio

R can be freely downloaded and installed from the R Project for Statistical Computing (https://www.r-project.org/). Additionally, users will download R Studio, a graphical interface for editing and running R packages freely available for download at https://www.rstudio.com. The Axoquant 2.0 script can be downloaded at https://github.com/BarkerLabUBC/Axoquant2.0 and opened in R Studio. Prior to analysis, images of DRG quarter-fields were organized in subfolders (one subfolder per well) named by embryo ID number, treatment name and repetition number within a single parent (experiment) folder. To analyze an experiment, the directory path to the experiment folder was entered in line 2 of the code designated “experiment.folder,” and the code was executed through to the end of the script. As images are processed, the R Studio console displays an ascending image count to indicate progress. When finished with analysis, Axoquant 2.0 automatically exports a *.csv data file to the parent folder that can be opened in spreadsheet and in statistical analysis software. For statistical testing, the data was binned in 500 μm increments by averaging axon density within these bins. Data was imported into GraphPad Prism 6 for statistical analysis with two-factor ANOVA (repeated measures in the distance factor) followed by Dunnett’s post hoc comparison with NGF-deprived controls (S1 and S2 Tables).

## Results and discussion

### Cultured DRG explants are imaged, cropped and organized for analysis

**Fig 1:**
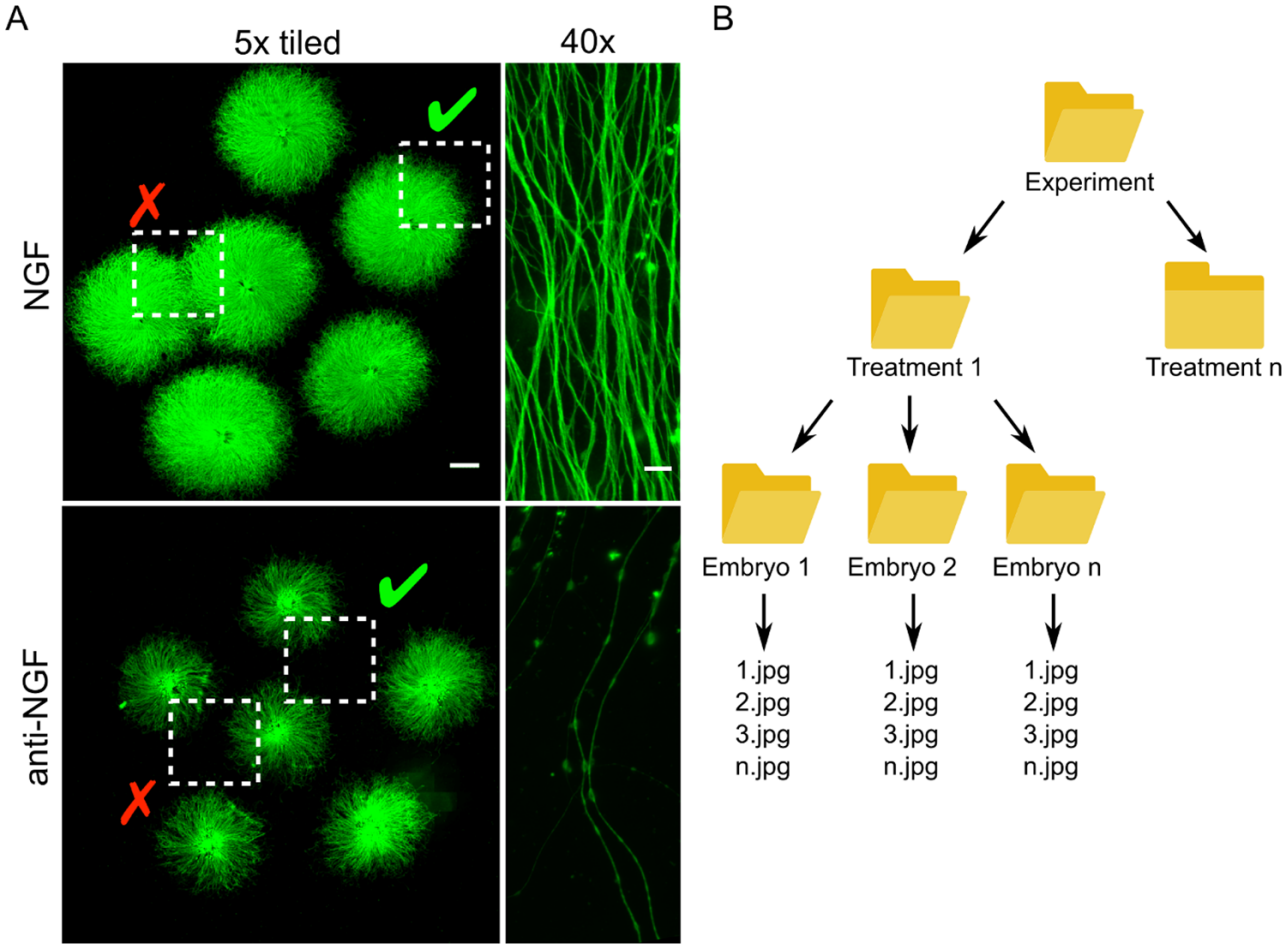
Axoquant 2.0 quantifies neurite degeneration from quarter-field images of DRG explants. DRG explants are dissected from E13.5 mouse embryos and seeded on 6-well plates (two wells shown in A; NGF (top) & anti-NGF 24h (bottom), scale bar = 1 mm for 5x images and 10 μm for 40x images). To quantify the degree of degeneration following a phase of NGF withdrawal and to assess the effect of pharmacological or genetic manipulations on its progression, the entire culture is imaged (after fixation and tubulin cytoskeletal immunostaining) by automatic tile-scanning on a motorized microscope stage, and quarter-fields are cropped and saved according to embryo and treatment (A; fields containing axons from only a single DRG are chosen, indicated by dotted box and green checkmark). The user directs Axoquant 2.0 to the experimental parent folder, where subfolders organized by treatment and embryo are crawled and quantified automatically (B).

In DRG cultures, axonal density decreases as distance from the ganglia increases and variations in the culture substrate can alter axon density within and between DRG. We established Axoquant 2.0 as a systematic and unbiased method for measuring axon integrity. To prepare images of axonal fields for input into Axoquant 2.0, the entire growth area on 6-well plates containing fixed and immunostained DRG explants are imaged via motorized microscope stage and images are stitched (Fig 1A). The user then crops and saves quarter-field regions of DRGs (indicated by a dashed-square “✓” in Fig 1A; examples regions disqualified for analysis because of interference from neighbouring DRG explants are indicated by a red checkmark “✕”). The R script is directed towards the parent experiment folder, within which the user has folders organized hierarchically by treatment and individual embryo (considered as the independent behaving unit n, Fig 1B). As many DRG explants as possible per embryo should be plated to reduce variability among embryos during analysis, and a minimum of three embryos per treatment must be utilized in order to move forward with statistical analyses of effect sizes.

### Automated Axoquant 2.0 workflow

**Fig 2:**
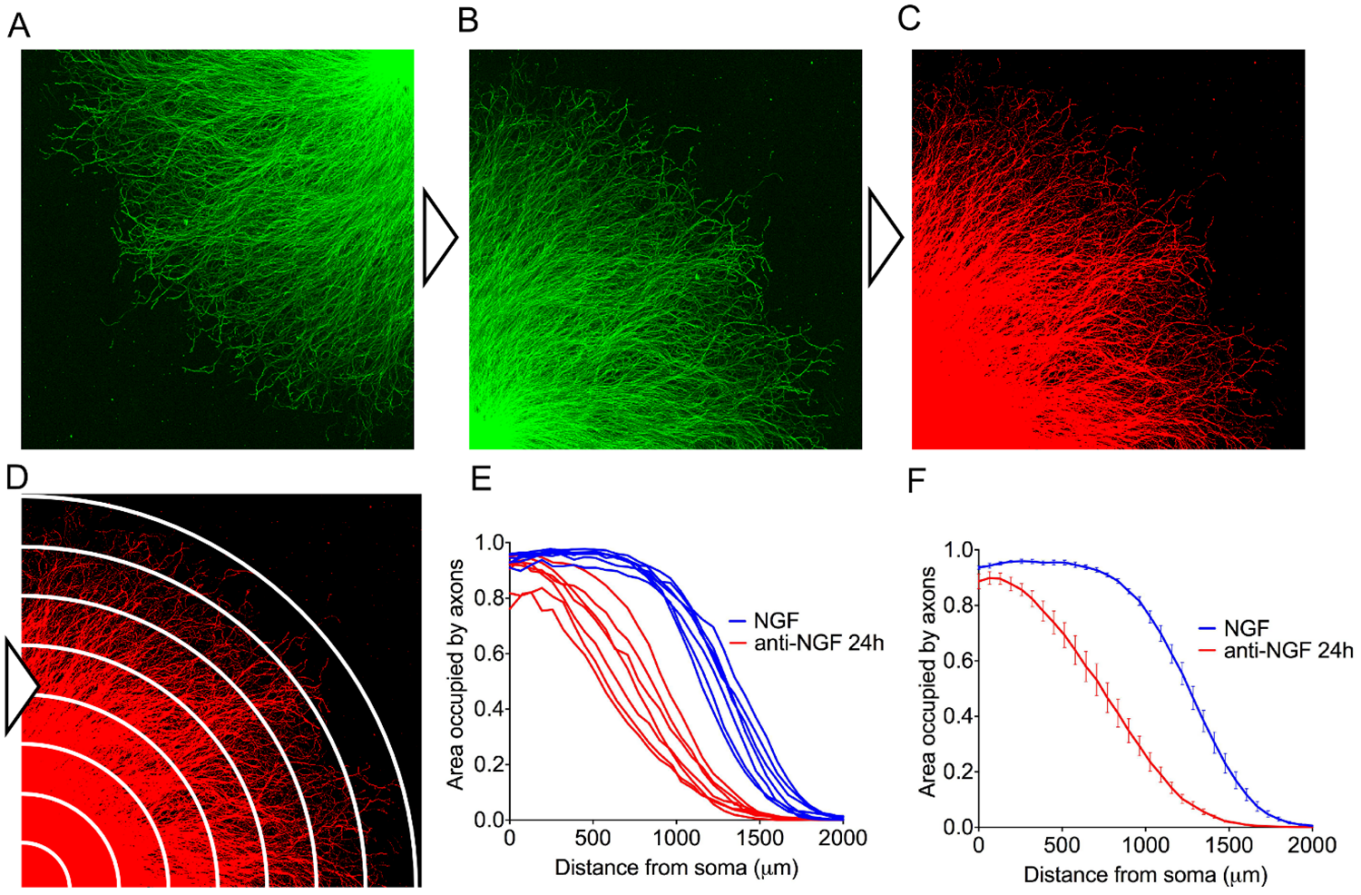
Axoquant 2.0 workflow. The R script automatically crawls folders and opens each quarter-field image (A) and if necessary, auto-orients the explant centre to the origin (B). Images are converted to binary masks with adaptive thresholding (C), and the area of the substrate occupied by axons is measured in bins radiating from the explant centre (stylized in D). Axoquant 2.0 automatically saves a comma separated file (*.csv) to the experiment parent folder for import into graphing and statistical analysis software. Example axon density curves are shown as embryo means (E) and treatment means with standard error (F).

The R script automatically detects the location of the neuronal cell bodies by identifying the most brightly stained corner, and each image is rotated to place the ganglion at the image origin (Fig 2A and 2B). A binary threshold is automatically applied to each image; by default the threshold is set at the mean pixel intensity of a given image plus 1.5 times the standard deviation of pixel intensity to create a mask of stained axons (Fig 2C). In our laboratory, the default threshold value delineates well-stained axons from substrate, but can be manually changed by users who wish to apply a higher or lower threshold to accommodate suboptimal stains. The density of binary-masked axons is then measured in 20 μm bins radiating outwards from the ganglia centre (Fig 2D). This approach may superficially resemble a Sholl analysis, which quantifies branching complexity in single neurons by counting intersections of dendrites with radially-drawn circles centred on cell bodies, but serves a fundamentally different purpose; Axoquant 2.0 utilizes radial bins to quantify axon density as a function of distance from soma, rather than branching complexity [16]. By default, 1 pixel is set to equal to 3.87 μm to process images generated by our system, but this value can be customized on line 183 of the script to accommodate images of other scales. Axoquant 2.0 automatically creates and saves a master comma separated file (*.csv) for import into spreadsheet software in the parent folder containing the experiment images. This data file contains individual measurements for each DRG, but also the mean of all DRGs within the same well, which is utilized for statistical analysis to compare treatment effects (Fig 2E). The axon density profile can be plotted as a function of distance from the soma in ganglia, and clearly reveals a rapid loss in axon density with distance, highlighting the imprudence of randomly sampling axons at multiple distances (Fig 2E and 2F).

### EGTA rescues axons from developmental degeneration *in vitro*

**Fig 3:**
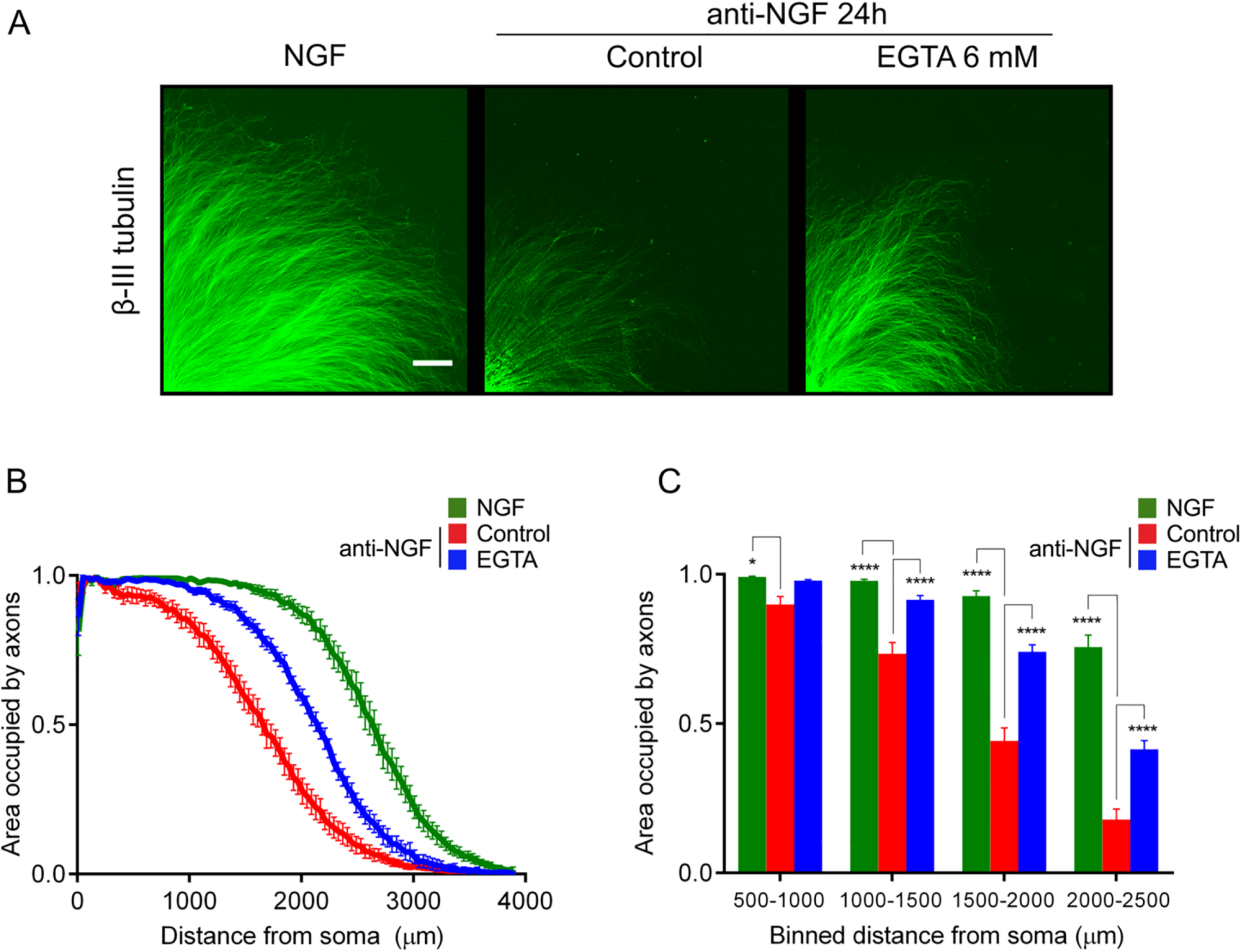
Axon degeneration following NGF withdrawal is rescued by Ca^2+^ chelator EGTA. (A) Explant ganglia were cultured in NGF and then either maintained in NGF (left, scale bar = 500 μm), deprived of NGF with a function-blocking anti-NGF antibody (middle), or deprived of NGF but in the presence of 6 mM EGTA (right). After 24 hours, cultures were fixed and immunostained with anti-ß-III tubulin primary antibody. (B) Axon density as a function of distance from the explant center was quantified by Axoquant 2.0 and (C) binned in 500 μm increments for statistical testing. NGF deprivation induced a significant loss of axons compared to NGF-supplied axons (p<0.05 within the bin at 500–1000 μm, and ps<0.0001 within the 1000–1500, 1500–2000, and 2000–2500 μm bins). Axons incubated with EGTA during the phase of NGF deprivation were significantly more dense than axons deprived of NGF without EGTA (ps<0.0001 within 1000–1500, 1500–2000 and 2000–2500 μm bins) indicating rescue of axons by Ca^2+^ chelation. n = 9 embryos per condition; plotted are mean and SEM. *p<0.05, ****p<0.0001, obtained by Dunnett’s post hoc comparison with anti-NGF control following two factor ANOVA, F (6, 72) = 37.87, p<0.0001.

Pathological Ca^2+^ stress is emerging as a key factor in diverse neurodegenerative disease states [17–21], and sporadic evidence suggests Ca^2+^ influx may be prodegenerative in NGF-deprived axons (8). Studies from more than 20 years ago examined the role of Ca^2+^ in the death of NGF-dependent neurons and concluded that Ca^2+^ does not play a significant role in degenerative signaling with these cells [22–24], prompting us to revisit this issue in DRG. Intriguingly, Fig 3 shows that the Ca^2+^ chelator ethylene glycol-bis(β-aminoethyl ether)-N,N,N',N'-tetraacetic acid (EGTA) rescues axons from degeneration. DRG cultures were either maintained in NGF (Fig 3A, left panel), deprived of NGF with a function-blocking anti-NGF antibody for 24 hours (middle), or deprived of NGF in the presence of EGTA (right panel). NGF deprivation for 24 hours induced robust axonal loss that was partially rescued by incubation with EGTA during the NGF withdrawal period (Fig 3A & 3B; binned for statistical testing in 3C). A two factor ANOVA (repeated measures in the distance factor) performed on the axon density indicated a significant effect of treatment, F (6, 72) = 37.87, p<0.0001, n = 9 embryos from 3 experiments. Dunnett’s post hoc comparison indicated that NGF deprivation induced a significant loss of axons compared to those supplied with NGF (p<0.05 within the bin at 500–1000 μm, and ps<0.0001 within the 1000–1500, 1500–2000, and 2000–2500 μm bins). Axons incubated with EGTA during the phase of NGF deprivation were significantly more dense than axons deprived of NGF in the absence of EGTA (all ps<0.0001 within the 1000–1500, 1500–2000 and 2000–2500 μm bins) indicating neuroprotection by Ca^2+^ chelation.

## Conclusions

NGF withdrawal from embryonic sensory neurons has been a valuable *in vitro* tool for dissecting prodegenerative signaling pathways for more than half a century, yet gaps remain in our mechanistic understanding of the sequence of events that unfold to destroy an axon. Objective, automated quantification of neurodegeneration is essential in the DRG system to test the effect of gene knockouts and chemical treatments on axon loss. Axoquant 2.0 accommodates the distinctive radial growth pattern of DRG axons to collect data at all distances from the neuron bodies, providing a global view of axon integrity, and negating the variability introduced by randomly sampling fields over regions that are intrinsically more or less dense as a function of outgrowth distance.

Early studies that failed to report Ca^2+^-dependent death of trophic factor-deprived sensory neurons utilized cultures maintained for up to 12 days. However, work published more recently has shown that sensitivity to NGF deprivation is reduced as embryonic cultures age beyond 2–3 days, and established that sensitivity is reduced in DRGs from older embryos [22–25]. Thus, the protective effect of Ca^2+^ chelation that we observed, versus the lack of effect in earlier studies, likely reflects sensitivity of the bioassays employed and the age of cultures. Our study has revealed that Ca^2+^ chelation provides clear and robust protection of the tubulin cytoskeleton during NGF withdrawal, consistent with sporadic evidence for a role for Ca^2+^-regulated mediators of degeneration such as calpastatin, an endogenous inhibitor of Ca^2+^-activated proteases in this process [26]. This provides validation of Axoquant 2.0 as a useful analytical tool and indicates that Ca^2+^ fluxes play a crucial role in developmental degeneration.

## Supporting information

S1 Table ANOVA and post hoc results. (PDF)

S2 Table Raw data. (PDF)

## References

[1] Saxena S, Caroni P. Mechanisms of axon degeneration: from development to disease. Prog Neurobiol [Internet]. 2007 10 [cited 2014 Nov 6];83(3):174–91. Available from: http://www.ncbi.nlm.nih.gov/pubmed/17822833 17822833 10.1016/j.pneurobio.2007.07.007

[2] Fischer LR, Glass JD. Axonal degeneration in motor neuron disease. Neurodegener Dis [Internet]. 2007 1 [cited 2014 Oct 29];4(6):431–42. Available from: http://www.ncbi.nlm.nih.gov/pubmed/17934327 17934327 10.1159/000107704

[3] Kanaan NM, Pigino GF, Brady ST, Lazarov O, Binder LI, Morfini GA. Axonal degeneration in Alzheimer’s disease: when signaling abnormalities meet the axonal transport system. Exp Neurol [Internet]. 2013 8 [cited 2014 Oct 27];246:44–53. Available from: http://www.pubmedcentral.nih.gov/articlerender.fcgi?artid=3465504&tool=pmcentrez&rendertype=abstract 22721767 10.1016/j.expneurol.2012.06.003 PMC3465504

[4] Vickers JC, King AE, Woodhouse A, Kirkcaldie MT, Staal JA, McCormack GH, et al Axonopathy and cytoskeletal disruption in degenerative diseases of the central nervous system. Brain Res Bull [Internet]. 2009 10 28 [cited 2014 Oct 31];80(4–5):217–23. Available from: http://www.ncbi.nlm.nih.gov/pubmed/19683034 19683034 10.1016/j.brainresbull.2009.08.004

[5] Deckwerth TL, Johnson EM. Neurotrophic factor deprivation-induced death. Ann N Y Acad Sci [Internet]. 1993 5 28 [cited 2014 Nov 6];679:121–31. Available from: http://www.ncbi.nlm.nih.gov/pubmed/8512180 8512180 10.1111/j.1749-6632.1993.tb18293.x

[6] Levi-Montalcini R, Booker B. Destruction of the sympathetic ganglia in mammals by an antiserum to a nerve-growth protein. Proc Natl Acad Sci [Internet]. 1960 3 1 [cited 2014 Nov 12];46(3):384–91. Available from: http://www.pnas.org/content/46/3/384.full?ijkey=087c79d81ac182f1a4600220a3c8eabee6520e13&keytype2=tf_ipsecsha 16578497 10.1073/pnas.46.3.384 PMC222845

[7] Oppenheim RW. Cell death during development of the nervous system. Annu Rev Neurosci [Internet]. 1991 1 [cited 2014 Nov 6];14:453–501. Available from: http://www.ncbi.nlm.nih.gov/pubmed/2031577 2031577 10.1146/annurev.ne.14.030191.002321

[8] Vogelbaum MA, Tong JX, Rich KM. Developmental regulation of apoptosis in dorsal root ganglion neurons. J Neurosci [Internet]. 1998 11 1 [cited 2014 Nov 9];18(21):8928–35. Available from: http://www.ncbi.nlm.nih.gov/pubmed/9786998 9786998 10.1523/JNEUROSCI.18-21-08928.1998 PMC6793517

[9] Simon DJ, Weimer RM, McLaughlin T, Kallop D, Stanger K, Yang J, et al A caspase cascade regulating developmental axon degeneration. J Neurosci [Internet]. 2012 12 5 [cited 2014 Jul 11];32(49):17540–53. Available from: http://www.jneurosci.org/content/32/49/17540 23223278 10.1523/JNEUROSCI.3012-12.2012 PMC3532512

[10] Schuldiner O, Yaron A. Mechanisms of developmental neurite pruning. Cell Mol Life Sci [Internet]. 2014 9 12 [cited 2014 Oct 24]; Available from: http://www.ncbi.nlm.nih.gov/pubmed/25213356 10.1007/s00018-014-1729-6 PMC5086088 25213356

[11] Delcroix J-D, Valletta JS, Wu C, Hunt SJ, Kowal AS, Mobley WC. NGF signaling in sensory neurons: evidence that early endosomes carry NGF retrograde signals. Neuron [Internet]. 2003 7 3 [cited 2014 Nov 9];39(1):69–84. Available from: http://www.ncbi.nlm.nih.gov/pubmed/12848933 12848933 10.1016/s0896-6273(03)00397-0

[12] Neukomm LJ, Freeman MR. Diverse cellular and molecular modes of axon degeneration. Trends Cell Biol [Internet]. 2014 9 [cited 2014 Oct 15];24(9):515–23. Available from: http://www.ncbi.nlm.nih.gov/pubmed/24780172 24780172 10.1016/j.tcb.2014.04.003 PMC4149811

[13] Levi-Montalcini R, Hamburger V. A diffusible agent of mouse sarcoma, producing hyperplasia of sympathetic ganglia and hyperneurotization of viscera in the chick embryo. J Exp Zool [Internet]. 1953 7 [cited 2014 Nov 12];123(2):233–87. Available from: http://doi.wiley.com/10.1002/jez.1401230203

[14] Levi-Montalcini R, Hamburger V. Selective growth stimulating effects of mouse sarcoma on the sensory and sympathetic nervous system of the chick embryo. J Exp Zool [Internet]. 1951 3 [cited 2014 Nov 12];116(2):321–61. Available from: http://doi.wiley.com/10.1002/jez.1401160206 14824426 10.1002/jez.1401160206

[15] Unsain N, Heard KN, Higgins JM, Barker PA. Production and Isolation of Axons from Sensory Neurons for Biochemical Analysis Using Porous Filters. J Vis Exp [Internet]. 2014 7 8 [cited 2017 Sep 21];(89):e51795–e51795. Available from: http://www.jove.com/video/51795/production-isolation-axons-from-sensory-neurons-for-biochemical 10.3791/51795 PMC4212973 25046441

[16] SHOLL DA. Dendritic organization in the neurons of the visual and motor cortices of the cat. J Anat [Internet]. 1953 10 [cited 2018 May 3];87(4):387–406. Available from: http://www.ncbi.nlm.nih.gov/pubmed/13117757 13117757 PMC1244622

[17] Sanabria-Castro A, Alvarado-Echeverría I, Monge-Bonilla C. Molecular Pathogenesis of Alzheimer’s Disease: An Update. Ann Neurosci [Internet]. 2017 5 [cited 2017 Nov 29];24(1):46–54. Available from: http://www.ncbi.nlm.nih.gov/pubmed/28588356 28588356 10.1159/000464422 PMC5448443

[18] Schampel A, Kuerten S. Danger: High Voltage—The Role of Voltage-Gated Calcium Channels in Central Nervous System Pathology. Cells [Internet]. 2017 11 15 [cited 2017 Nov 29];6(4):43 Available from: http://www.ncbi.nlm.nih.gov/pubmed/29140302 10.3390/cells6040043 PMC5755501 29140302

[19] Surmeier DJ, Halliday GM, Simuni T. Calcium, mitochondrial dysfunction and slowing the progression of Parkinson’s disease. Exp Neurol [Internet]. 2017 12 [cited 2017 Nov 29];298(Pt B):202–9. Available from: http://www.ncbi.nlm.nih.gov/pubmed/28780195 28780195 10.1016/j.expneurol.2017.08.001 PMC6037988

[20] Pchitskaya E, Popugaeva E, Bezprozvanny I. Calcium signaling and molecular mechanisms underlying neurodegenerative diseases. Cell Calcium [Internet]. 2018 3 1 [cited 2018 Mar 16];70:87–94. Available from: https://www.sciencedirect.com/science/article/pii/S0143416017300660 28728834 10.1016/j.ceca.2017.06.008 PMC5748019

[21] Prudent J, Zunino R, Sugiura A, Mattie S, Shore GC, McBride HM. MAPL SUMOylation of Drp1 Stabilizes an ER/Mitochondrial Platform Required for Cell Death. Mol Cell [Internet]. 2015 9 17 [cited 2016 Feb 15];59(6):941–55. Available from: http://www.ncbi.nlm.nih.gov/pubmed/26384664 26384664 10.1016/j.molcel.2015.08.001

[22] Tong JX, Eichler ME, Rich KM. Intracellular Calcium Levels Influence Apoptosis in Mature Sensory Neurons after Trophic Factor Deprivation. Exp Neurol [Internet]. 1996 3 [cited 2017 Nov 29];138(1):45–52. Available from: http://www.ncbi.nlm.nih.gov/pubmed/8593895 8593895 10.1006/exnr.1996.0045

[23] Eichler ME, Dubinsky JM, Tong J, Rich KM. The Ability of Diphenylpiperazines to Prevent Neuronal Death in Dorsal Root Ganglion Neurons In Vitro After Nerve Growth Factor Deprivation and In Vivo After Axotomy. J Neurochem. 1994; 10.1046/j.1471-4159.1994.62062148.x 8189223

[24] Eichler ME, Dubinsky JM, Rich KM. Relationship of intracellular calcium to dependence on nerve growth factor in dorsal root ganglion neurons in cell culture. J Neurochem [Internet]. 1992 1 [cited 2018 Mar 29];58(1):263–9. Available from: http://www.ncbi.nlm.nih.gov/pubmed/1727434 1727434 10.1111/j.1471-4159.1992.tb09305.x

[25] Unsain N, Higgins JM, Parker KN, Johnstone AD, Barker PA. XIAP regulates caspase activity in degenerating axons. Cell Rep [Internet]. 2013 8 29 [cited 2014 Oct 20];4(4):751–63. Available from: http://www.ncbi.nlm.nih.gov/pubmed/23954782 23954782 10.1016/j.celrep.2013.07.015

[26] Yang J, Weimer RM, Kallop D, Olsen O, Wu Z, Renier N, et al Regulation of axon degeneration after injury and in development by the endogenous calpain inhibitor calpastatin. Neuron [Internet]. 2013 12 4 [cited 2018 Mar 29];80(5):1175–89. Available from: http://www.ncbi.nlm.nih.gov/pubmed/24210906 24210906 10.1016/j.neuron.2013.08.034

