# Supplementary material for: A novel method for quantifying axon degeneration
Source: PLoS One. 2018 Jul 18;13(7):e0199570. doi: 10.1371/journal.pone.0199570 (PMC6051587; doi:10.1371/journal.pone.0199570)
Supplement: S1 Table — (PDF) [file pone.0199570.s001.pdf]

Table Analy	Data 3

Two-way R	Matching: Stacked
Alpha	0.05

Source of \	% of total v	P value	P value sur	Significant?
Interaction	9.359	< 0.0001	****	Yes
Distance fr	53.37	< 0.0001	****	Yes
Treatment	29.02	< 0.0001	****	Yes
Subjects (m	5.279	< 0.0001	****	Yes

ANOVA tab	SS	DF	MS	F (DFn, DF	P value
Interaction	0.7137	6	0.1189	F (6, 72) =	P < 0.0001
Distance fr	4.07	3	1.357	F (3, 72) =	P < 0.0001
Treatment	2.213	2	1.107	F (2, 24) =	P < 0.0001
Subjects (m	0.4026	24	0.01677	F (24, 72) =	P < 0.0001
Residual	0.2261	72	0.003141		

Number of	0

Within each row, compare columns (simple effects within rows)

Number of	4
Number of	2
Alpha	0.05

Dunnett's m	Mean Diff.	95% CI of d	Significant?	Summary
500-1000				
Control vs.	-0.08	-0.1657 to (	No	ns
Control vs.	-0.09226	-0.1779 to -	Yes	*
1000-1500				
Control vs.	-0.181	-0.2666 to -	Yes	****
Control vs.	-0.244	-0.3296 to -	Yes	****
1500-2000				
Control vs.	-0.298	-0.3836 to -	Yes	****
Control vs.	-0.485	-0.5706 to -	Yes	****
2000-2500				
Control vs.	-0.235	-0.3206 to -	Yes	****
Control vs.	-0.5771	-0.6628 to -	Yes	****

Test details	Mean 1	Mean 2	Mean Diff.	SE of diff.	N1	N2	q	DF

Row 1								
Control vs.	0.8988	0.9788	-0.08	0.03815	9	9	2.097	96
Control vs.	0.8988	0.991	-0.09226	0.03815	9	9	2.418	96
Row 2								
Control vs.	0.7339	0.9149	-0.181	0.03815	9	9	4.745	96
Control vs.	0.7339	0.9779	-0.244	0.03815	9	9	6.396	96
Row 3								
Control vs.	0.4424	0.7403	-0.298	0.03815	9	9	7.811	96
Control vs.	0.4424	0.9274	-0.485	0.03815	9	9	12.71	96
Row 4								
Control vs.	0.1787	0.4137	-0.235	0.03815	9	9	6.16	96
Control vs.	0.1787	0.7559	-0.5771	0.03815	9	9	15.13	96
