# Supplementary material for: A novel method for quantifying axon degeneration
Source: PLoS One. 2018 Jul 18;13(7):e0199570. doi: 10.1371/journal.pone.0199570 (PMC6051587; doi:10.1371/journal.pone.0199570)
Supplement: S2 Table — (PDF) [file pone.0199570.s002.pdf]

ance from s				EGTA				
10	0.966667	1	1	0.5	0.75	0.666667	1	1
30	0.983333	1	1	1	1	1	0.933333	1
50	1	0.952381	1	1	1	1	1	1
70	0.977778	1	1	1	1	1	1	1
90	1	0.97619	1	1	1	1	1	1
110	0.991667	1	1	0.75	1	1	1	1
130	0.977778	0.988095	1	1	1	1	0.966667	1
150	1	0.984127	0.982143	1	1	1	0.968889	0.972222
170	0.988889	0.986395	1	1	1	1	0.982828	0.977778
190	0.977143	0.97619	0.987013	1	1	1	0.960741	0.962963
210	0.97381	1	0.984127	1	1	1	0.982145	0.946032
230	0.975926	0.970522	1	1	1	1	0.970855	0.953704
250	0.977778	0.978836	1	0.972222	1	1	0.972393	0.968254
270	0.988889	0.97619	0.950397	1	1	1	0.975238	0.968254
290	0.992593	0.989947	0.953618	1	1	1	0.938112	0.987654
310	0.993333	0.973768	0.950835	1	1	1	0.964316	0.94246
330	0.968425	1	0.977891	1	1	1	0.967763	0.947531
350	0.974159	0.981955	0.979592	1	1	1	0.957631	0.920455
370	0.960691	0.983333	0.958163	1	1	1	0.955159	0.972222
390	0.995238	0.990079	0.97861	1	1	1	0.978297	0.943305
410	0.98022	0.995238	0.991597	1	1	0.966667	0.964003	0.953872
430	0.978502	0.989011	0.972549	1	1	1	0.965088	0.978788
450	0.991905	0.980615	1	1	1	1	0.989454	0.961111
470	0.969381	0.989011	1	1	1	1	0.956316	0.982194
490	0.986162	0.983035	0.992063	0.985294	0.985294	1	0.971111	0.977669
510	0.991363	0.98424	0.991597	0.980769	0.972953	1	0.963978	1
530	0.984682	0.986395	0.982653	0.984375	0.982143	1	0.980379	0.964436
550	0.994527	0.988818	1	0.984375	0.973684	1	0.970157	0.973545
570	0.984983	0.990816	0.982208	0.980769	0.9875	1	0.972666	0.97619
590	0.990476	0.992262	0.988571	1	0.988636	1	0.969906	0.986216
610	0.989538	1	0.984921	0.986111	0.99	1	0.983889	0.980368
630	0.993103	0.992857	0.988571	0.985294	0.98913	1	0.979361	1
650	0.987249	0.997494	0.989011	0.9875	0.968864	1	0.9714	0.965323
670	0.970343	0.994818	0.972931	1	0.989583	1	0.980504	0.988005
690	0.986155	0.998016	0.94907	1	0.988636	1	0.986829	0.987542
710	0.984063	0.994974	0.969928	0.988636	0.97151	1	0.965278	0.977381
730	0.985975	0.99793	0.974714	0.951786	0.98913	1	0.978814	0.979076
750	0.973127	0.99329	0.987578	0.97619	0.968652	1	0.963664	0.978676
770	0.985878	0.988152	0.959774	0.965368	0.983333	1	0.967917	0.987037
790	0.976621	0.998016	0.99038	0.939593	0.982809	1	0.964327	0.984031
810	0.959256	0.994303	0.967591	0.963636	0.991667	1	0.960063	0.995556
830	0.968742	0.998168	0.978475	0.964286	0.982759	1	0.939259	0.974143
850	0.958502	0.996032	0.959817	0.966667	0.991935	1	0.95003	0.973799
870	0.95804	0.991956	0.97379	0.948261	0.96133	1	0.921464	0.991587
890	0.955476	0.994159	0.952959	0.965909	0.965517	0.984127	0.955641	0.98449
910	0.971883	0.989337	0.971264	0.961071	0.946496	1	0.951671	0.960889

930	0.957907	0.992049	0.963005	0.95625	0.956303	1	0.955774	0.973783
950	0.963879	0.985861	0.941629	0.989583	0.964052	1	0.927061	0.97634
970	0.951806	0.984208	0.967865	0.962222	0.957821	1	0.954551	0.967186
990	0.945905	0.98859	0.932259	0.965446	0.945833	1	0.93028	0.961792
1010	0.94418	0.986447	0.961164	0.935185	0.958042	0.974359	0.923445	0.955466
1030	0.960245	0.983157	0.92934	0.933076	0.909521	1	0.914786	0.976014
1050	0.949209	0.988491	0.913465	0.916667	0.920762	1	0.91327	0.976386
1070	0.960927	0.982374	0.902625	0.929113	0.937339	1	0.931134	0.973336
1090	0.957856	0.981932	0.922313	0.905327	0.948622	1	0.909037	0.968345
1110	0.95102	0.984868	0.919026	0.899784	0.926565	0.987654	0.8851	0.972221
1130	0.934185	0.982788	0.933913	0.957143	0.933117	1	0.902021	0.95614
1150	0.934878	0.984308	0.914187	0.888341	0.92674	1	0.889327	0.954048
1170	0.923181	0.980947	0.90818	0.958991	0.943663	1	0.889404	0.947111
1190	0.921736	0.975424	0.908077	0.936333	0.905983	1	0.874051	0.958494
1210	0.919972	0.971764	0.87446	0.873168	0.8777	1	0.871898	0.960963
1230	0.925503	0.966894	0.882378	0.954768	0.897079	0.989247	0.867634	0.960121
1250	0.904685	0.961052	0.894794	0.917161	0.9425	1	0.871816	0.926268
1270	0.932726	0.969225	0.875135	0.939394	0.953426	0.979167	0.865451	0.916246
1290	0.901193	0.961001	0.84844	0.919492	0.926797	1	0.832183	0.910443
1310	0.883814	0.950401	0.839529	0.850673	0.926193	0.989247	0.840419	0.903033
1330	0.877586	0.950918	0.866526	0.891445	0.886714	0.971958	0.845573	0.942733
1350	0.889005	0.950764	0.854546	0.898923	0.933897	1	0.819896	0.912473
1370	0.885989	0.941421	0.846383	0.912626	0.886201	0.971148	0.827531	0.930877
1390	0.834796	0.944615	0.854425	0.865361	0.891226	0.939815	0.808333	0.911789
1410	0.883034	0.930891	0.856179	0.934375	0.902194	0.963186	0.814935	0.943543
1430	0.863241	0.918641	0.822266	0.875697	0.84881	0.974123	0.798654	0.902905
1450	0.858393	0.92998	0.844995	0.870039	0.875567	0.970274	0.749189	0.90648
1470	0.843174	0.925145	0.831236	0.811822	0.860701	0.982444	0.801337	0.891493
1490	0.788027	0.93386	0.824713	0.902778	0.846722	0.945574	0.747825	0.898867
1510	0.845272	0.916168	0.790993	0.826883	0.86952	0.92208	0.776633	0.905212
1530	0.818931	0.904271	0.791248	0.830958	0.834829	0.933312	0.756216	0.881081
1550	0.796459	0.888939	0.773704	0.846794	0.886046	0.973639	0.762633	0.877057
1570	0.807069	0.876464	0.796268	0.854065	0.83799	0.939624	0.735135	0.878426
1590	0.789633	0.890361	0.818688	0.842139	0.864512	0.95	0.709791	0.902238
1610	0.78035	0.868299	0.733891	0.849044	0.833013	0.92059	0.727365	0.878073
1630	0.7874	0.879165	0.775327	0.874229	0.851842	0.896051	0.683466	0.827997
1650	0.770919	0.855486	0.708195	0.78466	0.818465	0.945115	0.676282	0.831781
1670	0.785246	0.856147	0.764291	0.825589	0.801646	0.891414	0.696272	0.845312
1690	0.744403	0.835204	0.73256	0.796283	0.825199	0.85839	0.650587	0.790964
1710	0.725853	0.82284	0.702897	0.794854	0.785199	0.839267	0.638898	0.814481
1730	0.72837	0.832558	0.716824	0.818821	0.757079	0.873556	0.643304	0.799793
1750	0.716467	0.793438	0.714063	0.781597	0.785872	0.830476	0.616281	0.843599
1770	0.71743	0.802962	0.684644	0.753581	0.743728	0.855401	0.63801	0.784168
1790	0.696982	0.774526	0.658335	0.792379	0.755051	0.832653	0.598924	0.767484
1810	0.706411	0.765205	0.65746	0.759361	0.767722	0.803155	0.607356	0.759158
1830	0.68247	0.765232	0.594446	0.756106	0.737719	0.821999	0.590739	0.723736
1850	0.669049	0.727591	0.639858	0.760826	0.746325	0.849436	0.579118	0.751017

1870	0.637609	0.715359	0.585491	0.733495	0.717963	0.734501	0.566136	0.767638
1890	0.633911	0.702535	0.632459	0.740707	0.708766	0.740107	0.534794	0.720441
1910	0.629078	0.679699	0.582001	0.74468	0.716018	0.739049	0.56843	0.719601
1930	0.633521	0.674553	0.57586	0.684173	0.684246	0.705833	0.511075	0.703833
1950	0.612208	0.67088	0.517769	0.723068	0.652538	0.678917	0.479608	0.707414
1970	0.59476	0.633529	0.531991	0.676193	0.65682	0.675738	0.490646	0.652974
1990	0.551115	0.612358	0.562946	0.691289	0.64153	0.761961	0.497828	0.632686
2010	0.59455	0.599699	0.523246	0.660734	0.655259	0.667571	0.451398	0.666639
2030	0.534091	0.604012	0.526058	0.741479	0.643273	0.708357	0.46178	0.610444
2050	0.527704	0.574446	0.487298	0.656037	0.647501	0.662867	0.420232	0.622069
2070	0.517624	0.579588	0.454687	0.637514	0.63477	0.661368	0.410863	0.611993
2090	0.520376	0.522827	0.438822	0.639923	0.611205	0.594824	0.365237	0.59496
2110	0.522905	0.51989	0.436638	0.652004	0.557325	0.571593	0.396181	0.575828
2130	0.475839	0.509488	0.422119	0.636542	0.589735	0.622389	0.365269	0.547657
2150	0.486116	0.462132	0.418248	0.611842	0.607766	0.526091	0.349445	0.500143
2170	0.457177	0.457164	0.441249	0.568924	0.583306	0.536241	0.347204	0.527155
2190	0.447404	0.429044	0.346449	0.622555	0.537578	0.50776	0.325728	0.51387
2210	0.40889	0.415561	0.353257	0.616242	0.583938	0.519047	0.302763	0.49255
2230	0.391137	0.390923	0.341585	0.591406	0.507245	0.485346	0.322966	0.460292
2250	0.402696	0.38688	0.347546	0.579683	0.494357	0.459581	0.290712	0.49726
2270	0.353194	0.374165	0.277147	0.569419	0.461851	0.500312	0.281609	0.435341
2290	0.342361	0.352095	0.303944	0.498909	0.476329	0.381311	0.26092	0.43945
2310	0.344129	0.329718	0.260404	0.535994	0.463682	0.332265	0.263033	0.40475
2330	0.341317	0.304062	0.250822	0.492207	0.39347	0.385929	0.217442	0.425752
2350	0.299804	0.287117	0.220285	0.488248	0.391637	0.296075	0.212679	0.410582
2370	0.294431	0.278486	0.24257	0.485142	0.391724	0.378943	0.220669	0.354466
2390	0.249816	0.24935	0.216808	0.50844	0.360095	0.28618	0.219209	0.339438
2410	0.256273	0.241983	0.208352	0.459787	0.420984	0.239768	0.17495	0.379375
2430	0.248609	0.22201	0.195304	0.497631	0.43459	0.26649	0.183484	0.340742
2450	0.264852	0.204476	0.164684	0.4564	0.370728	0.25106	0.170212	0.339698
2470	0.227696	0.202114	0.198496	0.445633	0.341181	0.201241	0.161988	0.317927
2490	0.215633	0.190227	0.141313	0.443943	0.32526	0.200401	0.14519	0.285035
2510	0.194813	0.193311	0.146189	0.394191	0.296405	0.142415	0.155131	0.299888
2530	0.186403	0.179784	0.165964	0.395798	0.32127	0.165088	0.13574	0.279811
2550	0.200552	0.162854	0.139379	0.345721	0.327045	0.18332	0.148578	0.261235
2570	0.173202	0.153275	0.115635	0.39025	0.249138	0.163152	0.120293	0.248969
2590	0.162469	0.146863	0.097329	0.403156	0.30456	0.147536	0.126753	0.20687
2610	0.161424	0.139948	0.104559	0.379054	0.24544	0.137149	0.091723	0.24576
2630	0.139201	0.132836	0.122377	0.344981	0.244327	0.140415	0.109181	0.207344
2650	0.155955	0.130255	0.09884	0.356567	0.248409	0.110455	0.087316	0.230645
2670	0.146204	0.1218	0.103577	0.362131	0.227738	0.123098	0.071385	0.183838
2690	0.130131	0.088621	0.087895	0.305879	0.273254	0.113769	0.089662	0.174096
2710	0.109095	0.10075	0.093128	0.331035	0.175255	0.10757	0.077665	0.206511
2730	0.113959	0.076815	0.066246	0.338343	0.203559	0.05233	0.06854	0.165764
2750	0.112132	0.076305	0.059604	0.28866	0.203849	0.057287	0.076817	0.164287
2770	0.094741	0.067842	0.088408	0.343676	0.16132	0.03333	0.064462	0.141717
2790	0.080887	0.062329	0.075386	0.244596	0.15533	0.062805	0.06853	0.100445

2810	0.068727	0.059462	0.050708	0.302336	0.125479	0.038389	0.050797	0.126295
2830	0.082016	0.056257	0.051526	0.313325	0.129772	0.027778	0.056947	0.123041
2850	0.077962	0.050635	0.06663	0.298226	0.14946	0.036977	0.052021	0.095409
2870	0.0444	0.046491	0.050232	0.237293	0.135685	0.02254	0.038519	0.095744
2890	0.070116	0.041018	0.047234	0.266623	0.142061	0.035469	0.032824	0.103833
2910	0.047479	0.036216	0.040577	0.253259	0.09048	0.020526	0.037866	0.106281
2930	0.054385	0.03334	0.044639	0.275196	0.073077	0.018779	0.033639	0.08557
2950	0.037386	0.035907	0.027268	0.21134	0.058665	0.026967	0.027329	0.081254
2970	0.052697	0.032238	0.031197	0.314365	0.070189	0.013158	0.028595	0.095083
2990	0.048024	0.029928	0.018444	0.264065	0.066389	0.013894	0.027609	0.054026
3010	0.047708	0.030997	0.015792	0.232089	0.066932	0.013113	0.024624	0.063163
3030	0.035895	0.025113	0.014661	0.194624	0.047894	0	0.025511	0.041841
3050	0.033198	0.021029	0.006309	0.209623	0.020833	0.009148	0.013746	0.041679
3070	0.041951	0.025227	0.013516	0.193766	0.049206	0.008389	0.020859	0.047374
3090	0.021992	0.018208	0.009806	0.213429	0.032917	0.003968	0.009739	0.053096
3110	0.032062	0.018478	0.018095	0.169743	0.055804	0.004167	0.010477	0.033979
3130	0.022891	0.016607	0.014307	0.194722	0.024708	0	0.012872	0.042148
3150	0.027466	0.014517	0.012987	0.196288	0.023752	0	0.010968	0.032807
3170	0.021688	0.013939	0.006693	0.189568	0.007246	0.00844	0.002778	0.030855
3190	0.022782	0.012149	0.005425	0.172006	0.011059	0.008745	0.010489	0.026313
3210	0.017754	0.009161	0.0109	0.181444	0	0.004566	0.005239	0.028649
3230	0.015198	0.012668	0.012755	0.174736	0.015152	0	0.004566	0.018984
3250	0.01404	0.008425	0.00864	0.150538	0.027313	0.004016	0.005181	0.025781
3270	0.020278	0.008061	0.009204	0.170421	0.008621	0	0.003536	0.02308
3290	0.012939	0.007714	0.006122	0.10087	0.011538	0	0.002469	0.016883
3310	0.01495	0.011988	0.003663	0.166039	0.011018	0.003876	0.006625	0.014444
3330	0.01392	0.004978	0	0.119784	0.019949	0	0.001961	0.015816
3350	0.012058	0.009635	0.001661	0.13522	0.010521	0	0.006737	0.011241
3370	0.008631	0.006824	0.007534	0.13282	0.006966	0	0.007179	0.013338
3390	0.009567	0.009718	0.007376	0.132605	0	0.003546	0.002857	0.012644
3410	0.00695	0.003029	0.003574	0.073704	0.018452	0	0.00274	0.00433
3430	0.005141	0.005797	0.002268	0.104623	0.003676	0	0.006005	0.006292
3450	0.00757	0.004235	0	0.087947	0.007353	0	0.001852	0.011547
3470	0.011494	0.003284	0.004689	0.116757	0.005814	0	0.001026	0.00473
3490	0.006284	0.001591	0	0.086916	0	0.004386	0.001905	0.001634
3510	0.009291	0.002297	0	0.067073	0	0	0.00339	0.003131
3530	0.01026	0.004926	0	0.076667	0.009981	0	0.000926	0.001522
3550	0.007522	0.003735	0.002132	0.054598	0	0	0.001111	0.004533
3570	0.004182	0.004105	0.001786	0.097556	0	0	0.001042	0.00544
3590	0.008447	0.002801	0.006494	0.05102	0	0	0.002339	0.001764
3610	0.00117	0.002343	0	0.0625	0	0	0.00398	0
3630	0.004372	0.002614	0	0.062334	0	0	0	0.004665
3650	0.00119	0.001521	0	0.057054	0	0	0.001093	0.001406
3670	0.005178	0.002801	0.003968	0.054015	0	0	0.004678	0.001389
3690	0.002262	0.003124	0	0.028302	0	0	0.003279	0.004989
3710	0.001093	0	0	0.060204	0	0	0.003333	0
3730	0.005848	0.000866	0.002232	0.029677	0	0	0	0

3750	0.003407	0.001634	0	0.01875	0	0	0.002116	0
3770	0.003562	0.001752	0	0.053571	0	0	0.001149	0
3790	0.001149	0.000866	0	0.036252	0	0	0.001058	0
3810	0.00113	0	0.003759	0.051136	0	0	0	0
3830	0.00117	0.000821	0	0.031172	0	0	0.00226	0.001565
3850	0.002302	0.000934	0	0.046788	0	0	0	0
3870	0.004762	0.000835	0	0.053148	0	0	0	0
3890	0.002424	0	0	0.025771	0	0	0	0
3910	0.001111	0	0	0.028302	0	0	0	0

Control

0.857143	0.916667	0.85	1	0.714286	1	1	1	1
1	1	0.9	1	1	1	1	1	1
1	1	0.9	1	0.857143	1	0.9	1	1
1	1	1	1	1	1	1	1	1
0.952381	1	0.966667	0.97619	1	1	1	1	1
1	1	0.975	0.985714	1	1	1	0.969697	1
0.942857	1	0.966667	0.985714	1	1	1	1	0.974359
0.928571	1	0.955	0.971429	1	1	1	1	0.973626
1	1	0.966667	0.989796	1	1	0.985714	0.991736	0.980769
0.964286	0.991667	0.94	0.977891	1	1	1	1	0.966188
0.963203	0.980324	0.928889	0.973214	1	1	0.988889	1	0.970766
0.95273	0.980235	0.9375	0.922619	1	1	0.975	1	0.969475
0.925325	0.971991	0.852576	0.905471	1	1	0.983333	0.990909	0.97703
0.904762	0.987179	0.913889	0.945153	1	1	0.975	0.986513	0.975656
0.938591	0.976221	0.903571	0.936677	1	0.958333	0.992308	0.990909	0.980614
0.97619	0.96503	0.884237	0.919643	1	0.972222	0.986667	0.991736	0.966654
0.97114	0.927726	0.89	0.918615	1	0.863636	0.978571	0.993939	0.971335
0.960996	0.953312	0.873908	0.937771	1	0.909091	0.95625	0.979167	0.98109
0.954382	0.945182	0.860985	0.934637	1	0.81875	0.982353	1	0.996656
0.979592	0.934799	0.849874	0.955492	1	0.751894	0.964242	0.983094	0.97735
0.992857	0.948574	0.880592	0.89213	0.988095	0.754545	0.952851	0.986102	0.997151
0.981366	0.963176	0.869831	0.908635	1	0.777273	0.973333	0.995671	0.987701
0.985714	0.940904	0.861515	0.956052	0.988095	0.777778	0.95587	0.994318	0.992877
0.985103	0.970395	0.846734	0.920746	0.991071	0.758413	0.948785	0.98719	0.994302
0.983516	0.962609	0.895833	0.918239	0.988095	0.732143	0.962338	0.984848	0.981329
0.993506	0.975849	0.890313	0.902043	0.991071	0.757212	0.957857	0.991718	0.989698
0.968739	0.94799	0.85792	0.916929	1	0.765625	0.96006	0.98961	0.985254
0.989796	0.959399	0.891221	0.887078	0.987013	0.734375	0.95933	0.992576	0.995338
0.983516	0.957173	0.883523	0.901042	0.97551	0.803571	0.948901	0.992727	0.985483
0.989418	0.962812	0.850456	0.899381	0.991071	0.734375	0.954444	0.97583	0.972519
0.983654	0.948757	0.876814	0.891651	0.969925	0.75	0.960831	0.984305	0.990062
0.995238	0.95375	0.845884	0.870406	0.990476	0.719643	0.954142	0.988318	0.97606
1	0.96963	0.893297	0.893792	0.992063	0.777778	0.951905	0.968146	0.971152
1	0.926461	0.859358	0.845662	0.978571	0.770559	0.951381	0.975341	0.959529
0.982684	0.963382	0.885788	0.86918	0.977026	0.776042	0.939545	0.958793	0.965718
0.97619	0.919543	0.861869	0.87212	0.976608	0.74	0.943211	0.965869	0.979983
0.992063	0.930897	0.888894	0.87594	0.964502	0.771008	0.932346	0.954042	0.966774
0.979076	0.91597	0.820982	0.86532	0.984962	0.731115	0.942923	0.96415	0.974157
0.96281	0.887919	0.87495	0.831598	0.959802	0.763996	0.942289	0.961941	0.970192
0.97549	0.944875	0.811942	0.846229	0.975061	0.682407	0.935576	0.931255	0.960197
0.989229	0.903841	0.872186	0.853597	0.943182	0.678497	0.928535	0.925628	0.938275
0.954519	0.889868	0.835903	0.870754	0.973684	0.707776	0.960792	0.958184	0.9607
0.980788	0.887911	0.823197	0.844722	0.977566	0.717585	0.94025	0.953674	0.949203
0.978807	0.888646	0.847408	0.865445	0.979592	0.695076	0.931368	0.912708	0.950415
0.967878	0.855599	0.831049	0.847222	0.972217	0.666481	0.914455	0.926432	0.938787
0.97901	0.865007	0.838939	0.848669	0.975285	0.667852	0.919221	0.944595	0.957336

0.975872	0.889783	0.851118	0.79718	0.94375	0.62678	0.871516	0.924037	0.913139
0.970229	0.860303	0.830493	0.833455	0.961735	0.673326	0.902147	0.931603	0.917937
0.934157	0.865307	0.836318	0.807541	0.956889	0.638009	0.874931	0.922076	0.913171
0.960826	0.844108	0.790556	0.798076	0.957672	0.605929	0.873921	0.900439	0.914231
0.925358	0.840741	0.819245	0.824432	0.952977	0.606948	0.850602	0.914226	0.904501
0.950927	0.831091	0.821594	0.783952	0.970722	0.630367	0.895865	0.891334	0.907764
0.937341	0.821554	0.800832	0.806548	0.936223	0.58039	0.853164	0.888809	0.908915
0.936822	0.813782	0.796859	0.793878	0.928571	0.67134	0.826037	0.895222	0.901963
0.945639	0.79492	0.803356	0.814985	0.930973	0.516148	0.759387	0.908634	0.875807
0.943534	0.795303	0.794742	0.811973	0.935946	0.620459	0.794041	0.869754	0.896697
0.923504	0.826354	0.769907	0.795875	0.905925	0.518468	0.77599	0.874558	0.910868
0.950198	0.813038	0.780932	0.781543	0.879357	0.498696	0.743131	0.859501	0.876519
0.922736	0.803935	0.786652	0.774327	0.887081	0.580666	0.752192	0.855115	0.859903
0.914465	0.793919	0.787293	0.754563	0.892876	0.568817	0.756856	0.868988	0.89599
0.923781	0.77598	0.755711	0.787281	0.884905	0.500991	0.684663	0.848395	0.849378
0.919331	0.78933	0.781771	0.761692	0.912452	0.4938	0.657237	0.827966	0.847652
0.895682	0.740737	0.733813	0.745045	0.874433	0.516807	0.689565	0.826531	0.843843
0.86983	0.761895	0.735853	0.732107	0.840168	0.431399	0.671282	0.828725	0.827601
0.903098	0.741765	0.751905	0.731793	0.872535	0.453349	0.604035	0.818115	0.802428
0.892032	0.726733	0.761359	0.714381	0.794598	0.429638	0.606097	0.787319	0.77999
0.868733	0.740827	0.739807	0.713735	0.844117	0.443561	0.579693	0.768824	0.776903
0.871521	0.771841	0.745561	0.724513	0.747446	0.397018	0.613303	0.765248	0.784303
0.877379	0.716091	0.72548	0.722491	0.77524	0.377247	0.557173	0.742689	0.775311
0.806	0.703453	0.712362	0.703035	0.764996	0.418811	0.574654	0.736343	0.751615
0.817005	0.738505	0.699192	0.691909	0.779957	0.35373	0.529307	0.745868	0.717545
0.803034	0.679783	0.712226	0.698086	0.761791	0.358923	0.512004	0.714617	0.729074
0.82599	0.685442	0.721503	0.672937	0.714961	0.313203	0.520713	0.68081	0.703514
0.7897	0.656736	0.685625	0.658238	0.735383	0.308134	0.499142	0.708109	0.697256
0.817956	0.675049	0.676187	0.664476	0.705911	0.291199	0.473113	0.668258	0.696753
0.815465	0.682111	0.691485	0.666243	0.685005	0.30859	0.442648	0.638715	0.659511
0.804013	0.615823	0.670442	0.665518	0.669792	0.331704	0.446173	0.648133	0.665215
0.754394	0.65527	0.64865	0.645517	0.679909	0.317319	0.435356	0.616687	0.640389
0.748474	0.638875	0.641768	0.654116	0.68335	0.266377	0.372733	0.61006	0.630231
0.71472	0.606813	0.635243	0.634756	0.622884	0.343214	0.390682	0.604058	0.609971
0.741137	0.566357	0.67127	0.625525	0.681108	0.268548	0.405095	0.590032	0.62248
0.769112	0.580697	0.635529	0.596425	0.637929	0.249417	0.384019	0.587163	0.581518
0.719787	0.586266	0.644784	0.615433	0.555043	0.248626	0.383598	0.565024	0.569404
0.66718	0.546778	0.58002	0.620532	0.593738	0.246759	0.379029	0.569517	0.551315
0.724725	0.54017	0.602675	0.603822	0.539303	0.183145	0.340337	0.517672	0.520795
0.684792	0.567731	0.551188	0.589771	0.573331	0.201003	0.297021	0.490327	0.478184
0.710581	0.570596	0.563129	0.581927	0.529874	0.261844	0.324468	0.495598	0.471929
0.653617	0.542126	0.550533	0.560314	0.514867	0.186219	0.289005	0.480542	0.46935
0.651422	0.520402	0.557164	0.565559	0.565307	0.199822	0.313456	0.443261	0.462234
0.649168	0.513204	0.573569	0.544243	0.513649	0.145828	0.275418	0.419864	0.397852
0.637425	0.475202	0.569477	0.570167	0.467777	0.19558	0.285177	0.440468	0.425683
0.641771	0.443018	0.498779	0.559668	0.461122	0.173297	0.269952	0.438974	0.380358
0.62504	0.465994	0.504132	0.51246	0.493216	0.120112	0.272517	0.39481	0.368993

0.581505	0.431021	0.554986	0.533288	0.437256	0.153095	0.224674	0.363568	0.382945
0.558077	0.440636	0.508902	0.499433	0.468503	0.132092	0.267861	0.365988	0.319862
0.584301	0.387948	0.513404	0.510406	0.367331	0.118772	0.214689	0.317532	0.337893
0.577939	0.362582	0.497143	0.494469	0.37798	0.112197	0.231614	0.290448	0.320757
0.555908	0.386057	0.460273	0.47442	0.417939	0.103656	0.225736	0.289226	0.310262
0.533038	0.383364	0.458458	0.474596	0.396853	0.108969	0.20893	0.274843	0.292825
0.531055	0.379197	0.45968	0.455485	0.37185	0.072613	0.186418	0.256843	0.277835
0.50843	0.341639	0.46324	0.436557	0.330888	0.078715	0.177249	0.245235	0.262434
0.485913	0.326931	0.420611	0.452131	0.353333	0.106014	0.169154	0.244974	0.249049
0.495702	0.321291	0.410629	0.448091	0.308096	0.096365	0.15557	0.226861	0.229567
0.457146	0.284469	0.431959	0.413341	0.335272	0.071443	0.143944	0.209813	0.218086
0.459719	0.288352	0.392707	0.44403	0.314289	0.067707	0.150727	0.182331	0.216339
0.399628	0.29185	0.396969	0.378516	0.324646	0.05469	0.133106	0.177381	0.171411
0.421483	0.283891	0.365639	0.412497	0.279441	0.048088	0.136917	0.178437	0.184643
0.406803	0.2671	0.380982	0.363101	0.248806	0.082339	0.143295	0.154074	0.15435
0.393256	0.219864	0.404626	0.371619	0.3019	0.073365	0.149553	0.142765	0.155097
0.370604	0.270079	0.324731	0.358033	0.258775	0.082969	0.121134	0.120394	0.155704
0.389538	0.21084	0.304769	0.334096	0.262924	0.084459	0.10403	0.106058	0.124146
0.363122	0.220769	0.3596	0.344845	0.248962	0.063954	0.122347	0.107628	0.145151
0.327399	0.197651	0.30043	0.358696	0.221876	0.054324	0.088444	0.097245	0.123716
0.320608	0.198424	0.337078	0.33043	0.211453	0.049702	0.07922	0.08767	0.109493
0.298528	0.165373	0.294016	0.324838	0.22684	0.029788	0.094951	0.066567	0.117923
0.322857	0.168831	0.358439	0.306829	0.20767	0.049987	0.073221	0.074776	0.079008
0.287791	0.170137	0.289542	0.280944	0.200645	0.025054	0.073393	0.081664	0.11435
0.267336	0.146468	0.29149	0.291813	0.221278	0.032896	0.085383	0.068633	0.083914
0.262367	0.159741	0.306071	0.275523	0.193913	0.028511	0.062625	0.0534	0.077891
0.266053	0.130346	0.265535	0.251758	0.166723	0.026473	0.047242	0.03769	0.08055
0.242392	0.176708	0.25867	0.259523	0.169927	0.029796	0.076573	0.045018	0.06264
0.254094	0.113319	0.255103	0.270614	0.164235	0.022154	0.044582	0.042231	0.071059
0.224657	0.118183	0.225641	0.234705	0.168254	0.01722	0.051125	0.034024	0.063476
0.197632	0.099706	0.224318	0.231101	0.132192	0.014655	0.042801	0.035981	0.070092
0.197072	0.090018	0.25121	0.21169	0.129115	0.013158	0.072202	0.02046	0.050913
0.151865	0.077827	0.211654	0.202185	0.119431	0.010223	0.040863	0.020077	0.065022
0.15612	0.098423	0.217436	0.188424	0.159631	0.020097	0.049005	0.027799	0.043005
0.164603	0.090844	0.201494	0.204221	0.111856	0.008087	0.04339	0.023753	0.041461
0.159001	0.092163	0.176501	0.195973	0.091352	0.011062	0.035043	0.018364	0.033559
0.116999	0.068178	0.197075	0.180623	0.105088	0.01456	0.055326	0.018649	0.03066
0.124217	0.079618	0.186033	0.171135	0.109486	0.007249	0.043714	0.00955	0.035501
0.138717	0.058717	0.172994	0.17126	0.093729	0.011084	0.038473	0.010386	0.017461
0.10067	0.058983	0.159636	0.161165	0.110498	0	0.031886	0.006846	0.029448
0.10605	0.058462	0.141037	0.168892	0.096926	0.006849	0.036037	0.009859	0.022185
0.100713	0.065314	0.151236	0.151863	0.108366	0.011489	0.044495	0.006501	0.026768
0.103873	0.054467	0.138318	0.16326	0.07487	0	0.03185	0.008362	0.027569
0.083014	0.060351	0.134604	0.129177	0.071323	0.012035	0.030685	0.007385	0.019271
0.072271	0.047705	0.106814	0.122999	0.085355	0.013857	0.019494	0.012671	0.020354
0.099386	0.038942	0.10724	0.12101	0.094743	0.011661	0.035996	0.001151	0.017864
0.07032	0.03883	0.108463	0.129546	0.077952	0.006056	0.028417	0.003619	0.017167

0.078213	0.037	0.116201	0.093773	0.042003	0.007353	0.028695	0.006452	0.011034
0.073173	0.030619	0.104493	0.123429	0.059694	0.006448	0.021027	0.003691	0.02288
0.074323	0.033328	0.08613	0.095372	0.058047	0.003788	0.019076	0.002259	0.018194
0.058944	0.025636	0.075527	0.080557	0.046923	0.003289	0.02378	0.005604	0.011604
0.04055	0.018787	0.087682	0.081238	0.060925	0.014532	0.018973	0.003291	0.008726
0.052429	0.028904	0.083675	0.088776	0.062618	0.006946	0.0279	0.002035	0.011212
0.041608	0.023283	0.06347	0.081	0.025618	0	0.019622	0.005446	0.009834
0.033058	0.017866	0.083061	0.07215	0.026354	0.006696	0.025341	0.001196	0.008825
0.027869	0.013342	0.05906	0.072139	0.04408	0	0.023465	0.00202	0.004181
0.019223	0.015235	0.05736	0.06422	0.039656	0	0.024264	0.001082	0.002653
0.023819	0.009484	0.06662	0.085051	0.046771	0	0.021943	0.001166	0.008971
0.03882	0.010819	0.044137	0.081757	0.031427	0	0.018027	0	0.009038
0.033636	0.017185	0.056366	0.074429	0.022641	0.006494	0.021432	0.002066	0.002442
0.019324	0.017862	0.061481	0.059868	0.031083	0	0.012433	0.000988	0.003184
0.024023	0.011587	0.05266	0.064772	0.028473	0.003425	0.01129	0.002573	0.004566
0.015888	0.018987	0.044096	0.057111	0.01386	0	0.012165	0.001541	0.005043
0.021877	0.004524	0.051483	0.061551	0.033669	0	0.015899	0.002235	0.00236
0.021003	0.007591	0.038769	0.05855	0.020224	0.006098	0.015885	0	0.005464
0.02104	0.015699	0.042248	0.049771	0.01942	0	0.019149	0.002457	0.00595
0.023993	0.007693	0.034766	0.039451	0.029867	0.003012	0.015818	0.003547	0.000905
0.008202	0.011863	0.023919	0.051481	0.023111	0	0.008014	0	0.002389
0.009803	0.007149	0.028789	0.047295	0.007114	0.003165	0.012698	0	0.002285
0.007636	0.008761	0.035686	0.043954	0.017106	0.00987	0.009677	0.002479	0.001099
0.008163	0.005112	0.025295	0.047862	0.023188	0.003571	0.009091	0.001976	0.001221
0.019598	0.002669	0.036188	0.039561	0.008163	0	0.013088	0.004132	0.00104
0.009073	0.005364	0.032686	0.031992	0.020625	0	0.013663	0.001357	0.001241
0.008567	0	0.011049	0.039044	0.020778	0	0.017253	0.001181	0.002484
0.007427	0.006728	0.018293	0.038913	0.023825	0.003676	0.009677	0.003247	0.001099
0.006026	0.004831	0.012362	0.032372	0.009417	0.003906	0.013846	0.001299	0.003498
0.002304	0.001603	0.018093	0.025771	0.014669	0	0.014436	0	0
0.001984	0.002478	0.015406	0.034838	0.013455	0	0.012308	0	0.002336
0.004329	0.00278	0.009839	0.037516	0.010352	0	0.00743	0	0
0.009475	0.001042	0.011887	0.024118	0.01482	0	0.013636	0	0.002502
0	0.002317	0.016747	0.024498	0	0	0.002899	0	0
0	0	0.011807	0.033036	0.012485	0	0.00625	0	0
0.004698	0	0.008117	0.037171	0.00947	0	0.017647	0	0
0	0.001634	0.005589	0.025764	0.012229	0	0.004569	0	0.00135
0	0	0.007939	0.032708	0.012175	0	0.017647	0	0.001221
0	0.002405	0.006151	0.031269	0.008589	0	0.007692	0	0
0	0.002471	0.005326	0.028525	0	0	0.013846	0	0.001202
0.00207	0	0.002083	0.020921	0.010453	0	0.007937	0	0
0	0	0.007705	0.018732	0	0	0.011449	0	0
0	0	0.00311	0.018135	0.006487	0	0.012069	0	0.002608
0	0	0.002381	0.019758	0.004202	0	0.007143	0	0
0	0	0.00805	0.02326	0	0	0.014063	0	0
0	0	0.005833	0.023993	0.003663	0	0.00566	0	0
0	0	0.004738	0.010664	0.002976	0	0.015873	0	0

0	0	0	0.022783	0.003484	0	0.008475	0	0.003497
0.002101	0	0.003158	0.019977	0.010954	0	0.011538	0.003497	0
0	0	0.001786	0.024243	0.002646	0	0.003333	0	0
0	0.001667	0.005552	0.016836	0.003247	0	0.005769	0	0
0	0	0.003536	0.022392	0	0	0.013115	0	0
0	0.001437	0.003604	0.020057	0.003571	0	0.005922	0	0
0	0	0.005339	0.021709	0	0	0.014754	0	0
0	0	0.001563	0.004842	0.006324	0	0.007143	0	0.001538
0	0	0	0.020315	0	0	0.010204	0	0

NGF

1	0.5	0.571429	0.888889	1	0.923077	0.428571	1	1
1	0.75	0.714286	0.777778	1	0.769231	1	0.875	0.909091
1	0.9	1	1	1	1	0.857143	1	1
1	1	1	1	1	1	0.857143	1	1
1	1	1	0.972222	1	0.923077	1	1	1
1	1	1	1	1	1	1	1	1
0.97625	0.95	1	1	1	1	1	0.875	1
0.99	1	1	1	1	1	1	1	1
0.995833	1	1	1	1	1	1	0.975	1
0.974623	1	1	1	1	0.989011	1	1	0.954545
0.95035	0.946667	0.971429	1	1	1	1	1	1
0.958641	0.975	0.916667	1	1	1	1	0.943254	0.939394
0.932218	1	0.904762	1	1	0.989011	1	0.940476	1
0.966466	0.963333	0.928571	0.981481	1	1	1	0.975	0.960498
0.951554	0.969048	0.971429	0.981481	1	1	1	0.950893	0.976912
0.961154	0.876667	0.939909	1	1	0.990385	0.952381	0.951042	0.990909
0.920859	0.914762	0.941327	0.959436	1	1	1	0.894138	1
0.936825	0.98	0.961735	0.977778	1	0.991453	1	0.986111	0.988636
0.946867	0.983333	0.982143	0.962522	1	1	1	0.943254	0.991736
0.958476	1	0.984127	1	1	1	1	0.975	0.992424
0.961881	1	1	0.986111	1	0.991453	1	0.952991	1
0.976766	0.98	0.979592	1	1	1	0.979592	0.988636	0.988636
0.967656	1	1	0.971781	1	1	1	0.955754	1
0.962009	0.979167	0.982143	0.971781	1	1	1	0.970486	1
0.971839	0.985714	1	0.988889	1	1	1	0.977941	1
0.953429	0.98	0.987013	1	1	0.992308	1	0.97822	0.991736
0.952792	0.9875	1	1	1	1	1	0.983333	1
0.958903	0.979798	1	0.990741	1	0.992308	1	0.958081	0.986742
0.946804	0.988889	1	1	1	1	1	0.989583	1
0.945638	0.990909	1	1	1	1	1	0.964756	1
0.936219	1	1	1	0.977778	0.994872	1	0.992188	0.985431
0.937207	0.990909	1	0.984568	1	1	1	0.992188	0.994949
0.939075	1	1	0.992063	1	0.995726	1	0.973214	0.993007
0.926023	1	1	0.993464	0.983333	1	1	0.985417	0.994949
0.93585	1	1	1	0.991228	1	1	0.985417	0.990909
0.930854	1	1	1	1	0.994505	1	0.97835	1
0.925876	0.993333	1	1	0.992754	0.995475	1	1	1
0.922685	1	1	0.978395	0.986111	1	1	0.981283	0.996047
0.906527	1	1	1	0.977273	1	1	0.97903	0.984848
0.919541	0.981746	1	0.993056	0.984127	1	1	0.950807	0.978355
0.901544	0.993333	1	0.983796	0.992754	0.995726	1	0.985294	0.984838
0.897723	1	1	0.986111	0.986111	1	1	0.994792	0.977356
0.90464	0.984615	1	0.988225	0.977273	0.995192	1	0.993421	0.994949
0.876565	0.987778	0.990476	0.984656	0.972222	0.995192	1	0.986111	0.992095
0.899308	1	1	1	0.988095	0.995192	1	0.993056	0.98961
0.871162	0.99375	0.992063	0.988611	0.9614	1	1	0.988366	0.995671

0.872416	0.994118	1	0.986626	0.982143	0.995951	1	0.978374	0.983108
0.891641	0.981818	0.985338	0.994709	0.965397	1	1	0.982551	0.994949
0.883035	0.980085	1	0.992063	0.968254	0.996337	1	0.944422	0.987918
0.839317	0.994737	1	0.987654	0.977011	1	1	0.976301	0.978041
0.866007	0.993333	1	0.983188	0.980769	0.996337	1	0.989565	0.991386
0.850415	0.987868	0.982208	0.979521	0.987654	0.997151	1	0.974937	0.992088
0.82646	1	0.975724	0.990448	0.964957	0.992131	1	0.982903	0.985085
0.816917	0.983322	0.993789	1	0.956039	0.997253	1	0.976426	0.996212
0.812904	0.984524	0.986704	0.981755	0.952983	0.993311	1	0.95953	0.984792
0.803725	0.976856	1	0.978129	0.959091	0.992626	1	0.979565	0.971893
0.820291	0.98975	0.993789	0.979325	0.972222	0.989132	1	0.950837	0.977039
0.7823	0.995652	0.994048	0.980254	0.939136	0.988094	1	0.966731	0.997326
0.760829	0.996154	0.992481	0.971518	0.983074	0.990598	1	0.981324	0.983283
0.781881	1	0.994048	0.980344	0.969356	0.989043	1	0.975713	0.9868
0.740061	1	0.982857	0.982914	0.975397	0.991832	1	0.972773	0.982086
0.732893	1	0.994505	0.986169	0.956452	0.987881	1	0.972747	0.984286
0.720501	0.990476	0.989011	0.979984	0.976295	0.997041	1	0.981544	0.988307
0.702263	0.985389	0.971429	0.988529	0.969636	0.989441	1	0.970786	0.981302
0.703403	0.975464	0.982208	0.964839	0.994624	0.990667	1	0.983834	0.984632
0.683639	0.995	0.975061	0.978183	0.941212	0.984439	1	0.990968	0.972719
0.681616	0.985749	0.971228	0.965863	0.986486	0.98854	1	0.976254	0.974096
0.640625	1	0.974401	0.967659	0.957589	0.982357	1	0.938048	0.990236
0.644564	0.972741	0.974203	0.962222	0.955476	0.996923	1	0.965423	0.968313
0.628921	1	0.977822	0.962157	0.960317	0.987215	1	0.967497	0.977263
0.612041	0.992154	0.989403	0.947242	0.970811	0.987384	1	0.96832	0.980912
0.576003	0.973902	0.966939	0.95396	0.96808	0.988009	1	0.97262	0.977269
0.570079	0.995	0.980056	0.94673	0.963505	0.987109	1	0.954678	0.976461
0.550255	0.990693	0.972047	0.943319	0.953425	0.994905	1	0.952046	0.963328
0.516976	0.988558	0.975916	0.951892	0.954557	0.988955	1	0.956975	0.966934
0.517423	0.979467	0.971582	0.930972	0.955711	0.993092	1	0.955754	0.977807
0.499642	0.989372	0.964995	0.935241	0.951852	0.983765	1	0.950878	0.967455
0.49818	0.970615	0.979931	0.925509	0.94939	0.991125	1	0.946371	0.981689
0.453782	0.983201	0.969489	0.897178	0.968333	0.985852	1	0.943625	0.969039
0.453378	0.988708	0.976291	0.906295	0.946487	0.983904	1	0.909298	0.965487
0.426882	0.985277	0.950866	0.910471	0.968254	0.97861	1	0.960094	0.962494
0.417294	0.975254	0.980858	0.944033	0.943412	0.989091	1	0.951106	0.967459
0.415386	0.977835	0.968296	0.900928	0.961069	0.993407	1	0.895461	0.968403
0.383883	0.975677	0.960884	0.903081	0.967312	0.994242	1	0.924211	0.912846
0.367194	0.959465	0.928997	0.892122	0.965909	0.983136	1	0.915558	0.947653
0.376377	0.940876	0.967355	0.893034	0.937263	0.985541	0.993506	0.907394	0.943264
0.359694	0.957011	0.948621	0.903846	0.946769	0.989282	1	0.884914	0.947701
0.324224	0.955362	0.953487	0.875319	0.948567	0.977366	1	0.875072	0.92569
0.319573	0.944055	0.940951	0.8927	0.970442	0.984349	0.995074	0.877644	0.962179
0.307473	0.941851	0.94735	0.873622	0.925713	0.966307	0.994898	0.912759	0.926102
0.282065	0.928188	0.955441	0.853813	0.937537	0.969298	0.993789	0.893556	0.92616
0.279827	0.938255	0.951951	0.873568	0.928453	0.96421	0.993506	0.870652	0.89971
0.253914	0.936773	0.944684	0.854207	0.930084	0.969173	0.984962	0.840007	0.888503

0.265834	0.932634	0.960325	0.844937	0.890983	0.95297	0.988095	0.858976	0.920401
0.243188	0.919218	0.949473	0.877555	0.949284	0.956576	0.995238	0.827572	0.909865
0.237111	0.959315	0.940481	0.858566	0.942774	0.950205	0.993506	0.814972	0.898429
0.216974	0.930021	0.948778	0.850779	0.888521	0.953454	0.981911	0.809184	0.926332
0.196237	0.923499	0.929347	0.839515	0.912724	0.942747	0.983249	0.795773	0.881787
0.184986	0.900313	0.930569	0.848141	0.916387	0.945648	0.963961	0.790356	0.876021
0.191949	0.925525	0.957799	0.809143	0.878574	0.934704	0.984403	0.807954	0.834026
0.160252	0.922575	0.936532	0.836982	0.859855	0.939985	0.970168	0.770555	0.859372
0.152674	0.90536	0.941758	0.796576	0.873323	0.941014	0.978528	0.779079	0.841487
0.152186	0.896937	0.968311	0.809417	0.87309	0.9287	0.977134	0.76016	0.82766
0.162989	0.936764	0.930283	0.747606	0.857237	0.89391	0.951396	0.781689	0.82938
0.118645	0.8599	0.943902	0.81778	0.8441	0.930518	0.984085	0.734205	0.814191
0.115351	0.89045	0.932581	0.739737	0.816567	0.901356	0.969031	0.720012	0.815487
0.114327	0.854591	0.889216	0.756078	0.824513	0.903218	0.975084	0.68814	0.79338
0.102143	0.876247	0.91316	0.744484	0.792191	0.915529	0.950135	0.675356	0.7281
0.091045	0.877184	0.92258	0.780431	0.805436	0.89464	0.974275	0.678949	0.764978
0.090722	0.835122	0.904576	0.725954	0.722509	0.898263	0.947597	0.662796	0.746783
0.101859	0.856878	0.92881	0.726047	0.797257	0.853323	0.959429	0.660662	0.723081
0.084072	0.827218	0.876379	0.703601	0.749831	0.873153	0.963411	0.644432	0.715197
0.069457	0.821599	0.893752	0.67682	0.696165	0.852792	0.943041	0.664001	0.697834
0.072423	0.843933	0.884778	0.697509	0.731333	0.891603	0.962246	0.599961	0.645271
0.07792	0.789239	0.888524	0.65352	0.688691	0.827295	0.958377	0.590813	0.624158
0.063127	0.790714	0.881144	0.675367	0.688836	0.856937	0.953153	0.571432	0.610433
0.061382	0.789562	0.871665	0.695829	0.683235	0.80984	0.907322	0.598573	0.592335
0.059033	0.800926	0.86613	0.620073	0.653301	0.825002	0.920128	0.517181	0.608562
0.059082	0.736443	0.86528	0.677405	0.628086	0.785267	0.913144	0.524852	0.565793
0.048431	0.783268	0.850016	0.66134	0.620517	0.788652	0.896288	0.51152	0.543048
0.041899	0.741155	0.82805	0.624553	0.600384	0.726961	0.900094	0.542697	0.530663
0.044628	0.728514	0.823003	0.605134	0.598354	0.74817	0.902192	0.478461	0.505717
0.040299	0.726537	0.78931	0.608353	0.562859	0.724005	0.861534	0.44788	0.488579
0.045232	0.70722	0.807648	0.618792	0.56554	0.73168	0.85751	0.433608	0.481669
0.031437	0.70426	0.823138	0.60261	0.534873	0.736364	0.866342	0.395232	0.468604
0.032105	0.683063	0.774459	0.578209	0.527546	0.686699	0.869042	0.416372	0.415462
0.039066	0.717002	0.790553	0.528535	0.535485	0.658377	0.826364	0.359355	0.432194
0.02771	0.684635	0.738858	0.499544	0.50682	0.670406	0.779561	0.337209	0.388779
0.03073	0.6279	0.704554	0.521001	0.490378	0.604979	0.821152	0.28076	0.429661
0.026815	0.645513	0.689899	0.463469	0.425917	0.605745	0.812123	0.337475	0.397139
0.028281	0.643428	0.693133	0.483732	0.424609	0.58094	0.801312	0.320479	0.37529
0.031108	0.606567	0.648115	0.447639	0.427727	0.607487	0.760406	0.264793	0.357306
0.018526	0.623754	0.624863	0.469551	0.406305	0.549503	0.805271	0.258767	0.331628
0.028761	0.604547	0.626513	0.452717	0.39627	0.515065	0.769547	0.283397	0.320314
0.010967	0.546768	0.641807	0.426595	0.348306	0.483962	0.699872	0.262377	0.33186
0.017566	0.618022	0.591174	0.392527	0.344259	0.483054	0.706424	0.23011	0.34429
0.024453	0.544878	0.570069	0.407458	0.329311	0.402316	0.706621	0.209614	0.246169
0.015757	0.538847	0.563052	0.370629	0.326802	0.414919	0.702583	0.215928	0.255311
0.014794	0.513547	0.545976	0.398226	0.30953	0.407765	0.629513	0.189565	0.244619
0.0223	0.514141	0.528155	0.371702	0.285687	0.412185	0.613482	0.190371	0.251844

0.01171	0.530109	0.491779	0.330633	0.288884	0.377488	0.644355	0.152113	0.236814
0.013791	0.491125	0.471225	0.329509	0.296765	0.36616	0.594199	0.142749	0.253732
0.014141	0.541046	0.391978	0.318081	0.286841	0.343306	0.605099	0.143742	0.202832
0.009448	0.46618	0.445527	0.264583	0.222415	0.302158	0.585827	0.155195	0.218221
0.011823	0.446128	0.415936	0.297715	0.251103	0.290488	0.5779	0.133062	0.198978
0.012507	0.471195	0.396355	0.260553	0.203912	0.292282	0.561503	0.124503	0.161913
0.011073	0.44084	0.383107	0.292878	0.218152	0.25043	0.504634	0.132595	0.170074
0.013078	0.425916	0.30134	0.245704	0.223471	0.216562	0.487915	0.090614	0.179512
0.003308	0.416041	0.321201	0.25602	0.177187	0.22551	0.481528	0.109019	0.140752
0.00836	0.377715	0.287792	0.226531	0.191417	0.21798	0.500561	0.076085	0.14331
0.008808	0.371295	0.255999	0.251397	0.181836	0.190422	0.415028	0.073021	0.154299
0.00501	0.378953	0.336986	0.186593	0.145799	0.178179	0.386604	0.072598	0.127351
0.008777	0.306619	0.21763	0.214905	0.147583	0.15957	0.42045	0.047299	0.12753
0.007848	0.341835	0.249291	0.188285	0.160569	0.157131	0.377217	0.042652	0.105311
0.006588	0.328494	0.237882	0.17772	0.114052	0.141511	0.347153	0.051841	0.105272
0.004765	0.278531	0.200124	0.141036	0.111773	0.121456	0.347527	0.055308	0.103516
0.001796	0.311339	0.183558	0.175236	0.108422	0.102485	0.340346	0.03277	0.093414
0.006824	0.249049	0.17551	0.146277	0.105603	0.098975	0.328377	0.050982	0.074989
0.004163	0.228992	0.186754	0.181493	0.081993	0.091993	0.303555	0.06091	0.084588
0.005577	0.274296	0.142642	0.128476	0.114742	0.10363	0.25421	0.03079	0.081337
0.005488	0.21941	0.12893	0.146954	0.084315	0.090313	0.330063	0.019006	0.07372
0.006443	0.244094	0.149208	0.113998	0.087844	0.100264	0.247358	0.033546	0.063678
0.000847	0.213824	0.158687	0.125858	0.080601	0.06538	0.261619	0.021893	0.065515
0.005172	0.19889	0.137737	0.101657	0.059034	0.077762	0.275625	0.016532	0.054029
0	0.209487	0.133265	0.105147	0.075497	0.058002	0.198774	0.021459	0.049931
0.006061	0.189073	0.135644	0.092604	0.059805	0.070255	0.258057	0.019022	0.056363
0.000943	0.144822	0.093823	0.101663	0.04962	0.060027	0.231684	0.021304	0.047696
0.001515	0.183138	0.09941	0.083754	0.059261	0.033969	0.229049	0.019382	0.040652
0.004417	0.127466	0.120548	0.077431	0.048154	0.062208	0.170621	0.017337	0.032014
0.001699	0.142907	0.095594	0.091745	0.042191	0.036369	0.202884	0.00826	0.038725
0.00414	0.155957	0.085204	0.076924	0.028688	0.047009	0.157175	0.009606	0.027862
0.003485	0.12738	0.096341	0.08452	0.036073	0.027208	0.167044	0.014509	0.026762
0.001646	0.123184	0.059689	0.065738	0.032113	0.035762	0.15041	0.007354	0.025839
0.005681	0.121971	0.056171	0.073959	0.025389	0.023227	0.147896	0.009621	0.038383
0.003342	0.127947	0.058551	0.083976	0.019251	0.035961	0.143826	0.009194	0.027499
0.003379	0.101905	0.029756	0.063377	0.019692	0.017766	0.141391	0.003472	0.020464
0.002587	0.12943	0.037483	0.063775	0.023213	0.016268	0.134873	0.007319	0.020861
0.002907	0.100878	0.061117	0.057948	0.030185	0.020131	0.121786	0.005346	0.012027
0.000943	0.096499	0.041466	0.050925	0.023137	0.010565	0.1269	0.010924	0.020919
0.000962	0.075926	0.035159	0.056554	0.026226	0.019089	0.117358	0.003546	0.01541
0.002041	0.072337	0.03054	0.068562	0.028087	0.010656	0.091941	0	0.015224
0.004	0.070321	0.043745	0.04366	0.013571	0.011744	0.04241	0.001761	0.009563
0.001857	0.066589	0.033971	0.058073	0.003268	0.00787	0.091351	0.002049	0.012582
0.00283	0.070438	0.030035	0.050623	0.021269	0.006018	0.078002	0.002049	0.006163
0.002041	0.07786	0.015983	0.057292	0.005052	0.006048	0.075031	0.001563	0.007174
0.000714	0.05361	0.018807	0.031914	0.006061	0.011565	0.073401	0.003819	0.00714
0.000806	0.041766	0.010582	0.045963	0.002825	0.005574	0.064623	0.002315	0.002777

0	0.041094	0.014596	0.03086	0.005464	0.007485	0.063412	0.001894	0.001623
0.001064	0.02931	0.015739	0.035315	0.002924	0.007676	0.076933	0.002016	0.00469
0.000746	0.051076	0.015229	0.035829	0	0.008325	0.05575	0.003462	0.00303
0	0.037241	0.010445	0.035456	0.002976	0.006109	0.045903	0	0.004873
0.002	0.031468	0.015268	0.032184	0	0.001241	0.058962	0.001953	0
0.002326	0.027029	0.009745	0.029296	0.002732	0.003746	0.047114	0	0.001541
0.004864	0.044561	0.007303	0.021985	0.005664	0.003784	0.065568	0	0
0.001111	0.046916	0.01039	0.026952	0.012974	0.00262	0.044439	0	0.004088
0	0.029277	0.0073	0.023631	0.002976	0.003911	0.045124	0.001953	0

1			
1			
0.888889			
1			
1			
1			
1			
1			
1			
0.977778	bin centre		
0.949735	750	0.974779	0.99251
1	1250	0.905142	0.961492
0.984127	1750	0.714437	0.789751
0.967593	2250	0.388985	0.387498
0.986111			
0.948232			
0.975			
0.964899			
0.97601			
0.947924			
0.969848			
0.959259			
0.973765			
0.984046			
0.959972			
0.981352			
0.984127			
0.969697			
0.992593			
0.97619			
0.96814			
0.985119			
0.986883			
0.991453			
0.988173			
0.979521			
0.990079			
0.991453			
0.99537			
0.982456			
0.989198			
0.981592			
0.987542			
0.970899			
0.954072			
0.968076			

0.972816
0.986823
0.975397
0.960264
0.986074
0.962324
0.967886
0.971481
0.93641
0.985786
0.969299
0.936652
0.973784
0.961321
0.954821
0.972075
0.946243
0.927542
0.952174
0.925998
0.944775
0.931615
0.942415
0.901033
0.945336
0.924127
0.89913
0.921691
0.926516
0.886026
0.932658
0.899392
0.863405
0.894371
0.849258
0.891447
0.857004
0.865967
0.855251
0.876858
0.856871
0.826461
0.840589
0.813356
0.791369
0.819534
0.808867

0.785194
0.801447
0.786286
0.743884
0.768483
0.752969
0.744144
0.718234
0.748043
0.736191
0.709836
0.690896
0.722089
0.683834
0.638023
0.649395
0.636673
0.610759
0.643077
0.59447
0.552052
0.587401
0.563098
0.531761
0.491635
0.557239
0.49208
0.495028
0.453759
0.485855
0.435315
0.440633
0.431998
0.397292
0.429902
0.380248
0.36566
0.374166
0.327695
0.338843
0.350694
0.308966
0.29912
0.265373
0.266851
0.262044
0.237329

0.258297
0.229763
0.194567
0.208419
0.207428
0.170569
0.158825
0.151685
0.152547
0.134683
0.121413
0.13055
0.098124
0.1169
0.097621
0.111187
0.088365
0.091008
0.085707
0.092773
0.079046
0.074382
0.05859
0.082023
0.056848
0.061662
0.049457
0.044137
0.048794
0.04142
0.066861
0.038459
0.036633
0.035359
0.033482
0.032768
0.029703
0.026051
0.030791
0.029049
0.027466
0.0237
0.010941
0.02408
0.0109
0.018628
0.017665

0.008992
0.016076
0.020413
0.005366
0.011368
0.005368
0.014653
0.008896
0.007812

		EGTA						
0.972822	0.974164	0.975611	0.999365	0.961795	0.979496	0.97854	0.916591	0.854015
0.881132	0.907107	0.910643	0.985528	0.85577	0.938232	0.889264	0.761552	0.755991
0.681688	0.781671	0.771186	0.838891	0.629421	0.790647	0.665386	0.51553	0.569707
0.328693	0.563866	0.499392	0.44972	0.292847	0.467737	0.344805	0.210479	0.33256

		Control						
0.861401	0.974209	0.718201	0.934075	0.95576	0.959812	0.914991	0.992133	0.998196
0.746552	0.849182	0.475204	0.67117	0.811758	0.820884	0.713023	0.990103	0.983595
0.570164	0.532197	0.201952	0.314664	0.468374	0.469912	0.338899	0.952711	0.955152
0.335413	0.239258	0.052153	0.103952	0.113652	0.13484	0.086425	0.820104	0.886421

		NGF				
0.99299	0.986531	0.997551	1	0.980582	0.99102	0.980371
0.972246	0.965966	0.990735	1	0.970482	0.981362	0.94666
0.883783	0.939272	0.972722	0.993844	0.884766	0.93146	0.832444
0.70408	0.722723	0.847127	0.937666	0.61729	0.67287	0.594695
